# Integrating Clinical, Functional, and Patient-Reported Outcomes in Haemophilia Care: A Delphi-Based Consensus on a New Monitoring Tool

**DOI:** 10.3390/jcm15072533

**Published:** 2026-03-26

**Authors:** Angelo Claudio Molinari, Erminia Baldacci, Giovanni Barillari, Antonella Coluccia, Antonio Coppola, Anna Chiara Giuffrida, Gaetano Giuffrida, Chiara Gorio, Silvia Linari, Matteo Luciani, Alessandro Catini, Ilaria Nichele, Flora Peyvandi, Berardino Pollio, Annarita Tagliaferri, Federica Valeri, Maria Rosaria Villa, Ezio Zanon, Mariasanta Napolitano

**Affiliations:** 1 Thrombosis and Hemostasis Unit, IRCCS Istituto Giannina Gaslini, 16147 Genova, Italy; aclaudiomolinari@gaslini.org; 2 Haematology, Department of Translational and Precision Medicine, “Sapienza” University, 00185 Rome, Italy; baldacci@bce.uniroma1.it; 3 Center for Hemorrhagic and Thrombotic Diseases, General and University Hospital S. Maria della Misericordia, 33100 Udine, Italy; giovanni.barillari@asufc.sanita.fvg.it; 4 U.O.C di Medicina Interna, Centro Emofilia e Coagulopatie Rare-Ospedale “I.Veris delli Ponti”, Scorrano-ASL, 73010 Lecce, Italy; antonellacoluccia40@gmail.com; 5 Regional Hub Center for Hemophilia and Congenital Bleeding Disorders, University Hospital of Parma, 43126 Parma, Italy; acoppoladoc@gmail.com (A.C.); atagliaferri@ao.pr.it (A.T.); 6 U.O.C Medicina Trasfusionale, Azienda Ospedaliera Universitaria Integrata, 37126 Verona, Italy; annachiara.giuffrida@aovr.veneto.it; 7 U.O.C di Ematologia, A.O.U. Policlinico “Vittorio Emanuele”, 95123 Catania, Italy; gaegiuffrida@gmail.com; 8 Pediatric Onco-Hematology Unit, Children’s Hospital, ASST-Spedali Civili, 25123 Brescia, Italy; chiara.gorio@asst-spedalicivili.it; 9 Center for Bleeding Disorders and Coagulation, Department of Heart, Lung and Vessels, Careggi University Hospital, 50134 Florence, Italy; linaris@aou-careggi.toscana.it; 10 Hemostasis and Thrombosis Center, Onco-Hematology Department, Bambino Gesù Children’s Hospital, 00165 Rome, Italy; matteo.luciani@opbg.net; 11 Analisi Statistiche.it, 00149 Rome, Italy; a.catini@analisi-statistiche.it; 12 Centro Malattie Emorragiche e Trombotiche, UOC Ematologia, 36100 Vicenza, Italy; ilaria.nichele@aulss8.veneto.it; 13 Fondazione IRCCS Ca’ Granda Ospedale Maggiore Policlinico, Angelo Bianchi Bonomi Hemophilia and Thrombosis Center, 20122 Milan, Italy; flora.peyvandi@unimi.it; 14 Dipartimento di Fisiopatologia Medico-Chirurgica e dei Trapianti, Università Degli Studi di Milano, 20122 Milan, Italy; 15 SIMT Immunohematology and Transfusion Medicine Service, Diagnostic Department, A.O. Città della Salute e della Scienza, 10126 Turin, Italy; berardino.pollio@hotmail.it; 16 Regional Centre for Hemorrhagic and Thrombotic Diseases, Azienda Ospedaliera Universitaria Città della Salute e della Scienza,10126 Turin, Italy; fvaleri@cittadellasalute.to.it; 17 Hematology Unit, Ospedale Santa Maria di Loreto, 80142 Naples, Italy; mariarosariavilla@hotmail.com; 18 Haemophilia Centre, First Chair of Internal Medicine, Padua University Hospital, 35128 Padua, Italy; ezio.zanon@unipd.it; 19 Department of Health Promotion, Mother and Child Care (PROMISE), Internal Medicine and Medical Specialties, University of Palermo, 90127 Palermo, Italy; 20 Haematology and Rare Diseases Unit, Hospital “V. Cervello”, 90146 Palermo, Italy

**Keywords:** hemophilia A, hemophilia B, management, monitoring instrument, Delphi methodology, outcome assessment, patient-reported outcomes

## Abstract

**Background**: An appropriate and effective management of haemophilia is currently based on a multidimensional evaluation of treatment adequacy. Current clinical practice however is still lacking standardised tools able to combine clinical, functional, and patient-reported outcomes. In this study a structured Monitoring Tool for haemophilia A and B was developed and validated through a Delphi-based expert consensus process. This study represents an expert consensus-based validation of a monitoring framework, rather than a clinical validation in patient cohorts. The tool is intended for use by haemophilia treaters during routine follow-up visits to support structured treatment reassessment. Score categories reflect the need for clinical re-evaluation or potential treatment optimisation, rather than disease severity. **Methods**: Italian haemophilia specialists were asked to participate to a panel over a two-round Delphi process. Experts rated the relevance of several predefined clinical domains—pharmacokinetics, bleeding episodes, joint health, adherence and quality of life (QoL)—and the individual items within each domain for patients on prophylactic or on-demand treatment. Consensus was defined by responses within an interquartile range (IQR) < 8. Section and item weights and Likert-based scoring values were used to reach a composite score between 0 and 100. **Results**: Consensus was achieved for all domains and items across haemophilia types and treatments, prophylaxis and on demand (Haemophilia A: 16 and 12 participants; Haemophilia B: 12 and 9, respectively). With reference to prophylaxis domains, bleeding episodes received the highest domain weight (31–32%), followed by joint health (27–29%) and adherence/QoL (21–23%) and pharmacokinetics (18–19%). For on-demand treatment, pharmacokinetics was excluded; bleeding episodes (38–40%) and joint health (35–37%) remained dominant. At the item level, dynamic joint health indicators (HJHS and HEAD-US changes) and longitudinal QoL changes consistently received the highest weights. The final scoring system categorised results as Excellent (0–25), Suboptimal (26–50), Poor (51–75), or Critical (76–100). **Conclusions**: The Delphi-validated Monitoring Tools provide a structured, weighted, and clinically relevant framework for assessing treatment adequacy in haemophilia A and B across prophylactic and on-demand settings. These tools allow multidimensional outcome assessment and may support a more consistent, personalised therapeutic decision-making. A prospective validation of the tool in clinical cohorts is warranted.

## 1. Introduction

Haemophilia A and B are X-linked monogenic bleeding disorders caused by deficiencies in coagulation factor VIII (FVIII) and factor IX (FIX), respectively. The estimated prevalence at birth per 100,000 males is 24.6 cases for Haemophilia A and 5 cases for Haemophilia B [1,2,3].

Clinically, haemophilia is characterised by recurrent spontaneous or trauma-induced bleeding episodes, primarily affecting weight-bearing joints, skeletal muscles, and soft tissues [2]. Although rare, intracranial and retroperitoneal haemorrhages represent potentially life-threatening complications, particularly in individuals with severe disease [2,3]. The severity of the bleeding phenotype is classified as “severe,” “moderate,” or “mild,” based on residual endogenous factor levels of less than 1 IU/dL, between 1 and 5 IU/dL, and between 5 and 40 IU/dL, respectively [4].

If untreated recurrent bleeding, often manifesting as joint haemorrhages, leads to the progressive development of haemophilic arthropathy that, over time, might result in irreversible joint damage, substantial disability, and a significant decline in quality of life (QoL) [2,3].

Replacement therapy with exogenous FVIII and FIX remains the cornerstone of haemophilia management and is administered either on demand to control acute bleeds or prophylactically to prevent them. Long-term, continuous and early started prophylaxis regimens are currently the evidence-based standard of care in patients with severe haemophilia and in those with moderate disease but severe phenotype, aiming at preserving joint health and quality of life [4]. The management of prophylaxis in Haemophilia A and B has undergone a paradigm shift with the introduction of extended half-life (EHL) FVIII and FIX concentrates [5,6,7].

These products have enabled a more ambitious approach to prophylaxis, moving beyond the tradition goal of converting a severe phenotype to a moderate one. The introduction of these newer FVIII and FIX concentrates has made it feasible to maintain substantially higher trough levels during routine replacement therapy compared to what was achievable with standard half-life products [4]. As a result, these advanced therapies have significantly enhanced bleeding control while improving treatment adherence by reducing the frequency of required administrations [5,8]. Further, they enhanced patients’ quality of life (QoL) and promoted greater functional independence allowing for a more active lifestyle [9,10]. In recent years, haemophilia management has further evolved beyond conventional factor replacement therapy, with the introduction of non-replacement agents (e.g., bispecific antibodies) and the emergence of gene therapy approaches. These therapeutic innovations have shifted treatment goals from bleed reduction toward bleed elimination and long-term functional preservation, while also modifying monitoring needs and follow-up strategies [11,12,13,14]. As treatment paradigms diversify, assessment of treatment adequacy can no longer rely solely on factor levels or infusion frequency, but requires integrated evaluation of bleeding outcomes, joint status, functional measures, and patient-reported health status across different therapeutic mechanisms.

Despite these therapeutic advances, several persistent challenges remain in clinical practice. Early detection of suboptimal responses remains difficult, even in the context of pharmacokinetic-guided prophylaxis, due to the need for careful, ongoing interpretation and monitoring of individual pharmacokinetic data and continuous evaluation of clinical outcomes [15]. Furthermore, episodes such as subclinical joint bleedings and synovitis are difficult to detect but play an important role in the progression of haemophilic arthropathy [16,17]. Additionally, monitoring real-life adherence continues to pose significant challenges as accurate adherence tracking is difficult to implement consistently in routine care, despite its known association with improved clinical outcomes [18].

These issues are affected by the lack of standardized, practical tools to support the routine monitoring of treatment outcomes and guide evidence-based therapeutic decisions. While existing guidelines provide high-level recommendations, they are not accompanied by integrated, user-friendly instruments suitable for day-to-day clinical practice [4]. This fragmented approach limits the clinician’s ability to have a comprehensive view of treatment efficacy and patient well-being. Therefore, identifying a standardized set of clearly defined clinically detected and patient-relevant health outcomes represents a critical step toward value-based haemophilia care [19,20,21,22,23,24]. Further, structuring such domains into an integrated format would facilitate consistent, longitudinal assessment of patient status and enable more individualized, outcome-driven treatment strategies in real-world clinical practice.

Despite recent advances in haemophilia management and the quite common use of personalised prophylaxis strategies, routine clinical practice still lacks an integrated, user-friendly instrument capable of capturing clinical, functional, imaging-based and patient-reported outcomes within a single framework. Existing guidelines provide broad recommendations but do not operationalise them into practical monitoring tools suitable for longitudinal real-world use. This gap limits the ability of clinicians to detect early suboptimal response, to standardise treatment reassessment across centres, and to support value-based care models.

As a result, treatment adequacy is often judged through fragmented indicators rather than through a comprehensive, longitudinal evaluation. To date, no standardised instrument has operationalised these multidimensional outcomes into a single, weighted framework designed to support routine monitoring and treatment reassessment. This gap limits comparability across centres and hinders the implementation of value-based, outcome-driven haemophilia care. Although several validated instruments are available to assess specific domains of haemophilia care—including bleeding rates, joint status, imaging findings, pharmacokinetic parameters, and patient-reported outcomes—these tools were developed to measure individual constructs rather than to provide an integrated assessment of overall treatment adequacy [25,26,27,28,29]. Even recently introduced multidimensional instruments such as Hemo-FAST and Hemo-TEM focus on functional status or treatment burden, respectively, without combining clinical, structural, and patient-reported indicators into a unified, weighted framework [30,31]. Importantly, no currently available tool operationalises these domains into a composite index in which their relative importance is explicitly defined and standardised through expert consensus. As a result, treatment decisions remain dependent on parallel interpretation of heterogeneous indicators, with substantial variability across centres.

To address this need, we developed a consensus-based Monitoring Tool through a structured Delphi process among a panel of experts. The proposed Monitoring Tool represents a conceptual advance by formally integrating established outcome domains into a weighted composite structure, in which domain and item contributions are determined through a structured Delphi process. Rather than introducing new measures, this framework re-organises existing validated metrics into a standardised, longitudinal decision-support instrument designed to enhance comparability, reproducibility, and transparency in routine haemophilia management. By integrating clinical and patient-reported outcomes into a weighted, consensus-based scoring system, this tool seeks to deliver a holistic framework for real-time decision-making in haemophilia care.

## 2. Materials and Methods

### 2.1. Expert Panel

An expert panel was established, composed of 8 members: 7 Italian clinicians with extensive expertise in haemophilia care (proven by reputation, published papers, presentations at national and international scientific meetings, and participation in clinical trials and expert panels) working in haemophilia centres with at least 40 registered severe PWH and a methodological expert in consensus-building processes, specifically the Delphi technique. This group was responsible for defining the clinical domains, developing questionnaire items, supervising data analysis, and validating consensus thresholds.

### 2.2. Questionnaire Development

The Monitoring Tool for Haemophilia A and B was developed through a structured co-creation process. Items were designed as single-choice questions using ordinal or Likert-type scales (three- or four-point). The tool captured key clinical domains including pharmacokinetics, bleeding episodes, joint health, as well as treatment adherence and health-related QoL. For each clinical domain one or more questions associated with an outcome were developed. A complete list of questions is provided in Table 1 and Table 2. Two distinct questionnaires were created to reflect the clinical context of patients receiving prophylactic and on-demand treatment, respectively. Clinical domains and outcomes were selected based on an extensive review of international guidelines and current literature and subsequently refined and scored through the Delphi consensus process [4,26,32,33,34,35,36,37,38,39].

More specifically, the identification of domains and items followed a structured pre-Delphi phase designed to minimise selection bias and enhance reproducibility. First, an exploratory mapping of outcome domains was conducted based on international haemophilia management guidelines (WFH, national consensus documents) and previously published outcome frameworks. Domains were screened for relevance to routine monitoring rather than for research endpoints alone. Candidate domains were required to meet three predefined criteria: (i) clinical relevance for treatment adequacy assessment; (ii) measurability using instruments validated or routinely applied in haemophilia care; (iii) and feasibility of collection during standard follow-up visits without requiring additional specialised procedures. Domains that were considered exploratory, highly specialised, or not routinely measurable in standard care settings were not retained for the preliminary instrument, pharmacokinetic (PK) adequacy was operationalised as a structured clinician-judgement item (“satisfaction with achieved trough level”) rather than a fixed numerical threshold. This choice reflects the heterogeneity of prophylaxis goals across patients and products (standard vs. extended half-life) and the fact that trough interpretation depends on bleeding phenotype, joint status, lifestyle, and regimen characteristics. Accordingly, the PK item captures perceived adequacy of exposure relative to individualised treatment targets.

Following this mapping phase, the expert panel independently reviewed the candidate domains and associated variables. Through structured discussion, items were refined to ensure clarity, actionability, and avoidance of redundancy. No weighting decisions were made at this stage. The resulting preliminary questionnaire was then submitted to the formal Delphi rounds for consensus-based weighting.

### 2.3. Participants in the Delphi Process

Experts invited to participate were 20 haemophilia specialists with at least three years of clinical experience in Haemophilia Centres homogeneously allocated on the national territory with at least 25 severe PWH registered. Overlap existed between the 8-member expert panel who designed the questionnaire and the Delphi participants due to limited availability of key opinion leaders in the field. However, item weighting decisions were based exclusively on anonymised Delphi responses, and no item-level weighting was predetermined during the questionnaire development phase. Invitations were sent to clinicians identified through national society rosters and publication records. Participation was voluntary. Completion rates decreased between Round 1 and Round 2, consistent with typical Delphi processes. The expert panel involved in tool development was distinct from the Delphi participants, although partial overlap occurred; roles were clearly defined to minimise anchoring bias. The expert panel was drawn from across the Italian national territory, ensuring broad and balanced geographic and institutional representation.

### 2.4. Delphi Rounds and Feedback

A classical Delphi methodology was employed to assess the clinical importance of each clinical domain (assigned a section weight, W) and individual question (assigned an item weight, w). For each clinical domain and individual question, participants rated its relevance as a percentage weight. The Delphi rounds were conducted online. Among the 20 invited experts, 16 participants completed the first round and 12 completed the second round in the Haemophilia A arm. In the Haemophilia B arm, the respective numbers were 12 and 9. Attrition between rounds was not formally investigated; however, non-participation in Round 2 was attributed to logistical constraints (e.g., clinical workload) rather than methodological disagreement.

In Round 1, experts were free to allocate weights, with the constraint that the total across clinical domains and questions had to equal 100. In Round 2, participants were asked to reassess their responses within the interquartile range (IQR) from the first round (i.e., between the first and third quartiles). If a score fell outside the IQR, written justification was required.

### 2.5. Consensus and Stopping Criteria

In this study, weights were elicited using a constant-sum approach: each expert distributed a total of 100 points across the items (with the sum constrained to 100), yielding weights on a 0–100 scale interpretable as relative percentage importance.

Convergence among expert ratings was assessed using the interquartile range (IQR), a robust measure of dispersion frequently employed in Delphi studies. Consensus was defined *a priori* as an IQR < 8 points for each item, corresponding to a dispersion of the central 50% of responses below 8% of the total budget. This deliberately stringent threshold was chosen to identify a high level of agreement on item weights. The threshold was specified prior to data collection and was not modified during or after the Delphi rounds.

The selected cut-off is consistent with examples from Delphi studies using 0–100 scales, in which thresholds of IQR ≤ 10 are associated with high levels of consensus (e.g., classified as “great consensus” in Alfaro Martínez et al., 2020), and is more conservative than other criteria reported in the literature for 0–100 metrics (e.g., IQR ≤ 25) [40]. The selected cut-off therefore represents a comparatively conservative threshold appropriate for percentage-based constant-sum weighting.

### 2.6. Data Analysis

Responses were analysed using a hierarchical weighted scoring algorithm. For each item, the final contribution to the composite score was calculated by multiplying the domain weight (W), the item weight within that domain (w), and the standardised Likert-scale response value (r). Likert responses were normalised as proportions on a 0–1 scale (four-point scale: 0, 0.33, 0.67, 1; three-point scale: 0, 0.5, 1), ensuring compatibility with the constant-sum weighting structure. The total composite score was obtained as the sum of all weighted item contributions and rescaled to a 0–100 range for interpretability. This multiplicative framework reflects the underlying clinical rationale that overall treatment adequacy is determined jointly by the relative importance of each domain, the relevance of individual items, and the patient’s status within each item, thereby allowing clinically critical domains to exert proportionally greater influence while preserving granular patient-level information.

To enhance clinical readability, the continuous 0–100 composite score was subsequently mapped onto four ordinal categories (0–25: Excellent; 26–50: Suboptimal; 51–75: Poor; 76–100: Critical). These cut-offs were defined during the development phase as evenly distributed intervals across the 0–100 scale, providing a transparent and pragmatic interpretative framework consistent with the percentage-based weighting structure. The categories were conceived as descriptive anchors reflecting increasing deviation from conditions considered optimal by expert consensus. They were not derived from associations with clinical outcomes and therefore do not represent prognostic or decision-making thresholds. Rather, they provide a preliminary classification intended to facilitate structured interpretation of the monitoring profile, pending prospective validation and empirical calibration against relevant clinical endpoints.

A fully worked numerical example illustrating step-by-step score computation and clinical interpretation is provided Supplementary Material S1.

### 2.7. Ethical Considerations

The process followed national ethical standards applicable to expert consultation and non-interventional research. All participants received information about the aims and methodology prior to participation.

## 3. Results

### 3.1. Development of the Monitoring Tool

In order to support a structured, multidimensional assessment of treatment adequacy in both prophylaxis and on-demand settings, the architecture of the Haemophilia Monitoring Tool was developed considering four clinical domains: pharmacokinetics, bleedings, joint health, as well as adherence and health-related QoL. The Expert Panel included in each domain specific, clinically validated variables aimed at capturing both the biological and experiential dimensions of haemophilia management. The selection of variables was guided by international guidelines, expert consensus, and the requirement that each item be measurable, relevant, and actionable in routine care [4].

In the pharmacokinetics domain, clinicians are asked to evaluate the satisfaction with the achieved trough level based not only on objective factor concentrations but also on the individual patient’s protection needs and the characteristics of the prophylaxis regimen. This reflects the shift toward personalised prophylaxis and underscores the importance of interpreting pharmacokinetic data in the frame of the individual clinical context [32].

The bleeding episodes domain is assessed through the annualised bleeding rate (ABR), calculated as the number of bleeding events divided by the duration of follow-up and multiplied by 365.25 [33]. For joint-specific bleeding, the tool also includes the Annualized Joint Bleeding Rate (AJBR), offering a more granular view of musculoskeletal vulnerability [33].

The joint health domain is comprehensively addressed through a combination of static and dynamic indicators. Clinicians are asked to record the current number of Problem Joints (PJ), defined as joints with chronic pain and/or limited range of motion due to structural compromise, and indicate whether these have improved, remained stable, or worsened since current treatment began [34]. A similar approach is applied to Target Joints (TJ), defined by frequency of spontaneous bleeds [35]. Functional and structural joint status is further assessed via changes in the Haemophilia Joint Health Score (HJHS) (i.e., clinical examination) and the Haemophilia Early Arthropathy Detection with Ultrasound (HEAD-US) score (i.e., ultrasound imaging), allowing the clinician to capture both visible and subclinical onset or progression of arthropathy [36,37].

For the adherence assessment, clinicians should estimate the patient’s treatment compliance, calculated as the ratio between administered and expected prophylactic infusions. This allows for a standardised, quantifiable approach to a behaviourally driven outcome often difficult to track consistently [38]. Finally, the QoL domain incorporates the EQ-5D-5L Level Sum Score, a validated instrument measuring health status across five dimensions [39]. Clinicians are asked to assess both the current QoL score and the change over time, reflecting not only baseline patient well-being but also its trajectory in response to treatment.

A complete list of questions with related answers developed for rating during the Delphi rounds is provided in Table 1 and Table 2 for patients on prophylaxis and on-demand treatment, respectively. A flow diagram summarising the Delphi process, including invitations, participation, and attrition between rounds, is provided in the Supplementary Material S3.

### 3.2. Domain and Item-Level Consensus

#### 3.2.1. Domain-Level Weighting Across Haemophilia Types

In patients receiving prophylaxis, consensus was achieved across all predefined domains in both haemophilia A and haemophilia B (Table 3 and Table 4). Domain weighting patterns were highly consistent between the two conditions.

Bleeding episodes received the highest weighting in both haemophilia B (32%) and haemophilia A (31%), reflecting its central role as the most immediate and tangible indicator of treatment efficacy. Joint health followed closely (27% in haemophilia B; 29% in haemophilia A), reflecting the enduring relevance of musculoskeletal integrity as a long-term outcome, even in well-managed patients.

The adherence and QoL domain was consistently assigned intermediate weight (23% in haemophilia B; 21% in haemophilia A), highlighting the recognized relevance of behavioural and patient-reported dimensions in optimizing treatment outcomes. Pharmacokinetics received comparatively lower weighting (18% in haemophilia B; 19% in haemophilia A), indicating that while clinically relevant, it was considered secondary to bleeding and joint-related indicators in the overall evaluation framework. Importantly, differences between haemophilia A and B were minimal and did not alter the hierarchical ranking of domains.

In the on-demand context, the pharmacokinetics domain was excluded in both haemophilia types, given its limited clinical applicability in episodic treatment models (Table 5 and Table 6).

Bleeding episodes emerged as the dominant domain (40% in haemophilia B; 38% in haemophilia A), followed closely by joint health (35% and 37%, respectively). The adherence and QoL domain consistently accounted for approximately one quarter of the total weight (25% in both haemophilia types). Overall, domain-level patterns in the on-demand setting mirrored those observed in prophylaxis, with strong concordance between haemophilia A and B.

#### 3.2.2. Item-Level Weighting

Within the prophylaxis setting, item-level analysis demonstrated a high degree of agreement among panellists across both haemophilia types (Table 7 and Table 8).

Within the joint health domain, the number and evolution of PJ and TJ were consistently weighted within a comparable range (11–15% per item in Haemophilia A; 13–14% in Haemophilia B). Changes in joint status assessed through clinical (HJHS) and instrumental (HEAD-US) measures were also prioritised. In haemophilia B, the HEAD-US score received slightly greater relative emphasis (19%), while in haemophilia A clinical and ultrasound changes were similarly weighted (15–17%). The annualised joint bleeding rate (AJBR) maintained stable relevance across both types (13%).

Within the adherence and QoL domain, adherence to prophylaxis accounted for approximately one third of the domain weight (32–33%). Current QoL received comparable importance (32%), while perceived change in QoL over time was slightly prioritised (35–36%), reflecting the panel’s emphasis on longitudinal patient-reported outcomes.

In the on-demand subgroup, joint-related items remained central across both haemophilia types (Table 9 and Table 10). The number of PJ and TJ were assigned similar weights (18–19%). Changes in joint status (HJHS and HEAD-US) were weighted prominently, with slightly higher emphasis on clinical and imaging evolution compared to prophylaxis settings. The AJBR retained consistent relevance (17–18%) across haemophilia A and B.

Across treatment settings and haemophilia types, weighting patterns were structurally coherent. Bleeding episodes and joint health consistently represented the core domains of evaluation, while adherence and QoL contributed meaningful complementary information. Pharmacokinetics was recognised as relevant in prophylaxis but not in on-demand management. The high degree of concordance between haemophilia A and B supports the internal consistency of the Monitoring Tool framework.

### 3.3. Definition of Final Scoring System

The Monitoring Tool’s scoring system was constructed as a weighted sum of the selected responses. Each response was weighted according to the section weight (W) (i.e., clinical domain), the specific item weight (w) (i.e., question domain), and the response option’s position on the Likert scale. For four-point Likert scales, values were defined as 0%, 33%, 67%, and 100%; for three-point scales, values were 0%, 50%, and 100%.

The final composite score ranges from 0 to 100 and serves as an indicator of the clinical adequacy of the patient’s current treatment. Scores between 0 and 25 are considered Excellent, indicating that no therapeutic changes are necessary. Scores between 26 and 50 are defined as Suboptimal, suggesting that treatment reassessment may be beneficial. Scores between 51 and 75 reflect a poor-quality therapeutic profile, and treatment re-evaluation is advised. Scores between 76 and 100 are categorized as Critical, signifying that the current management is likely inadequate and should be promptly revised.

## 4. Discussion

The present study establishes content validity through expert consensus, providing a structured and clinically grounded framework for monitoring treatment adequacy in haemophilia. It does not represent a clinical validation in patient cohorts, which will require prospective application and outcome-based assessment. Through a two-round Delphi process, involving a panel of Italian haemophilia experts, consensus was adequately reached across all the proposed domains and items, confirming the feasibility of defining a shared clinical framework for outcome evaluation [4,20,35].

Across different haemophilia types and treatments, experts reported a consistent prioritisation of outcome domains. In prophylaxis, bleeding episodes received the highest weight, reflecting their established role as the primary expression of therapy efficacy [33,41]. Joint health closely followed, mirroring the well-known burden of progressive arthropathy and the importance of an early detection of subclinical joint deterioration [16,34,36,37]. Adherence and QoL were also evaluated as crucial elements of a comprehensive care, in accordance with emerging evidence on the impact of adherence on bleeding outcomes and treatment efficacy [10,18,38].

Pharmacokinetics, though assigned a lower weight, was also considered relevant in the prophylaxis tool due to its contribution to an individualised treatment, mainly for EHL products and personalised prophylaxis [5,7,15,25,32].

For on-demand therapy, experts did not consider the pharmacokinetics domain, which is clinically non-applicable, and attributed the highest weights to bleeding episodes and joint health, mirroring a well-established priority for episodic treatments [34,35].

Item-level analysis supported a clear inclination for dynamic indicators of disease evolution. Changes in HJHS and HEAD-US scores were prioritised, supporting a recognised value of sensitive tools for detecting early arthropathic changes and synovitis [16,36,37]. Similarly, within the QoL domain, experts assigned the highest weight to QoL change over time, reflecting the increasing emphasis on patient-reported outcomes in haemophilia care [20,39,42].

The Monitoring Tools developed in the current study mirrors the outcome domains proposed in recent initiatives aimed at standardising haemophilia outcome evaluation [1,19,21]. Their added value lies in embedding these domains within a weighted, consensus-based scoring system, supporting clinicians in a well-structured, assessment [22,24].

The Delphi method allowed anonymity, well-structured feedback, and geographical representation across Italian haemophilia centres. These features reduce hierarchical bias and strengthen the generalisability of an expert consensus.

Several limits must also be addressed. Although the Delphi method is well suited to formalizing informed consensus in areas where objective criteria are not yet established, the size of the expert panel, particularly in the second round, and the limited availability of experts for haemophilia B may reduce the stability of the derived weights and their generalizability across settings. Smaller panels may increase the relative influence of individual ratings, and attrition between rounds may affect consensus distribution. Nevertheless, the narrow interquartile ranges observed across domains and items suggest a high degree of convergence among participants, supporting internal consistency of the weighting structure. Accordingly, the weights obtained from this Delphi exercise should be interpreted as evidence of content validity based on expert consensus rather than as statistically generalisable parameters. In addition, the national composition of the panel may limit applicability across different healthcare systems. Further studies are warranted to strengthen the external validity of the tool, including validation in real-world contexts and/or through larger, multi-context expert panels. Finally, given that the score categories are consensus-based and not calibrated against clinical outcomes, their interpretation should be considered preliminary. Prospective validation studies are required to assess the relationship between scores and/or score categories and relevant clinical endpoints, and to refine and optimize cut-offs (e.g., through analyses of discriminative accuracy and calibration) before the tool can be used as an outcome-validated decision-support instrument.

From an implementation perspective, the tool is designed to be completed during routine follow-up visits using routinely collected clinical data. Completion time is expected to be limited (under 10 min), provided that joint assessments and patient-reported outcomes are already part of standard care, thus making it easy to use. Training requirements are aligned with existing competencies for HJHS and HEAD-US assessment. In real-world settings, missing data may be addressed through clinical judgement, acknowledging this as a potential source of variability. Potential floor effects should also be considered. In well-controlled patients with ABR = 0 and no reported target or problem joints (TJ = 0; PJ = 0), variability in the composite score may be reduced, potentially limiting sensitivity at the lower end of the scale. However, the inclusion of structural and inflammatory indicators—such as synovitis detection through HJHS or HEAD-US—is intended to identify early joint changes that may not yet be reflected in bleeding frequency [43]. This design feature may partially mitigate floor compression, although empirical assessment of floor effects will be required in prospective validation studies. Regarding the score interpretation, elevated composite scores should be understood as structured clinical signals rather than automatic therapeutic mandates. A high score indicates potential misalignment between current management and the consensus-defined optimal profile and should prompt comprehensive clinical reassessment. Depending on the specific domain drivers, this may include review of prophylaxis adequacy, adherence evaluation, pharmacokinetic reassessment, imaging follow-up, or consideration of alternative therapeutic strategies. The Monitoring Tool is intended to support structured periodic evaluation within routine care rather than to dictate predefined treatment actions.

Despite these design features, feasibility may vary across healthcare infrastructures. The Monitoring Tool assumes availability of routinely collected clinical data and, in its current form, incorporates ultrasound-based joint assessment (HEAD-US), which may not be consistently accessible in low-resource settings or in centers without trained operators. In such contexts, reliance on clinical joint assessment alone could reduce sensitivity to early subclinical arthropathy and limit comparability across sites. Furthermore, integration into routine visits may increase clinician workload, particularly in high-volume clinics or systems with limited staffing. Finally, the PK domain relies on clinician judgement rather than a universal trough target, which may introduce inter-rater variability across centers. Prospective validation should therefore assess agreement and reliability of this item and explore whether additional guidance (e.g., goal-based anchors) improves consistency without undermining individualisation. These contextual factors should be considered in future implementation studies, including evaluation of minimum feasible datasets and setting-specific adaptations.

Future research should focus on prospective validation in real-world, multicentre cohorts. A first step could involve longitudinal application of the Monitoring Tool in patients undergoing routine follow-up (e.g., 12–24 months), with evaluation of its association with established clinical endpoints such as annualised bleeding rate (ABR), annualised joint bleeding rate (AJBR), changes in HJHS and HEAD-US scores, and patient-reported quality-of-life measures. Discriminative accuracy analyses (e.g., ability of baseline scores or categories to identify patients requiring treatment modification), calibration against clinician global assessment, and responsiveness to therapeutic changes (e.g., transition from standard to extended half-life prophylaxis or initiation of non-replacement therapy) should be assessed. Comparative evaluation against standard-of-care monitoring approaches will be essential to determine incremental clinical utility. Such studies would allow empirical refinement of score thresholds and establish the tool as an outcome-validated decision-support framework [19,42].

## Figures and Tables

**Table 1:** Overview of the questions to be rated during the Delphi process for patients on prophylaxis.

Clinical Domain	Question with Related Answers	Definition
Pharmacokinetic	Based on the patient’s needs and the current prophylaxis regimen, how satisfactory is the obtained trough level? Not at all satisfactory; Not very satisfactory; Quite satisfactory; Very satisfactory	The trough level refers to the lowest concentration of clotting factors VIII or IX present in the patient’s plasma immediately before the next scheduled infusion. The assessment of satisfaction with the achieved trough level is based on the specialist’s evaluation, considering not only the objective numerical value, but also the patient’s individual need for protection (considering their specific characteristics) and the type of prophylactic regimen being used [32]
Bleeding episodes	What is the annualized bleeding rate (ABR)? Total ABR 0; Total ABR 1–2; Total ABR 3–4; Total ABR ≥ 5	ABR is calculated as the number of bleeding events divided by the duration of follow-up and multiplied by 365.25 [33]
Joint health	How many Problem Joints (PJs) does the patient currently present with? None; 1; 2–3; ≥4	A PJ is a joint with chronic pain and/or limited range of motion due to compromised joint integrity (e.g., chronic synovitis and/or hemophilic arthropathy), with or without persistent bleeding [34]
	Since the start of the current treatment, the PJs have Absent or decreased; Remained stable; Increased	
	How many Target Joints (TJs) does the patient currently present with? None; 1; ≥2	A TJ is a joint that has experienced three or more spontaneous bleeding episodes within a six-month period [35]
	Since the start of the current treatment, the TJs have: Absent or decreased; Remained stable; Increased	
	Since the last assessment, how has the clinical evaluation of the patient’s joint status changed? Improved or remained approximately unchanged (at most +1 point); Moderately worsened (+2/+3 points); Significantly worsened (+4 points)	Clinical evaluation is based on the difference between the initial total Haemophilia Joint Health Score (HJHS) and the current total HJHS. The HJHS is a clinical assessment tool designed to detect early signs of haemophilic arthropathy. It is based on the evaluation of joint damage in the elbows, knees, and ankles, and includes both structural and functional components [37]
	Since the last assessment, how has the ultrasound evaluation of the patient’s joint status changed? Improved or remained unchanged; Moderately worsened (+1 point); Significantly worsened (+2 points or more)	Ultrasound evaluation is based on the difference between the initial total Haemophilia Early Arthropathy Detection with Ultrasound (HEAD-US) score and the current total HEAD-US score. The HEAD-US score is a semi-quantitative imaging tool designed to detect and monitor early joint changes in people with haemophilia using point-of-care musculoskeletal ultrasound. The HEAD-US system provides a simplified scale for detecting and quantifying early joint damage affecting the synovium, cartilage, and subchondral bone in the elbows, knees, and ankles. It allows for the evaluation of joint status and the monitoring of its progression as an indicator of arthropathy in patients with haemophilia [36]
	What is the annualized joint bleeding rate (AJBR)? Total AJBR 0; Total AJBR 1; Total AJBR 2; Total AJBR ≥ 3	The AJBR is calculated as the number of reported joint bleeding events divided by the duration of follow-up and multiplied by 365.25 [33]
Adherence and QoL	How would you rate your patient’s adherence to the prophylactic treatment? Very satisfactory (>90%); Satisfactory (between 80% and 90%); Poorly satisfactory (<80%)	Adherence is calculated as: ([Number of prophylactic infusions during the treatment period] ÷ [Expected number of prophylactic infusions during the treatment period based on the prescribed regimen]) × 100 [38]
	How would you assess your patient’s current Quality of Life (QoL)? Very satisfactory (5–6 points); Moderately satisfactory (7–11 points); Poorly satisfactory (12–18 points); Not at all satisfactory (19–25 points)	Patient’s QoL is measured using the EQ-5D-5L (LSS) questionnaire. The EQ-5D-5L is a self-reported questionnaire that evaluates health status across five dimensions: mobility, self-care, usual activities, pain/discomfort, anxiety/depression. Each dimension has 5 levels of severity. The LSS is calculated by adding the numeric values from each of the five dimensions. The total score ranges from 5 (best health) to 25 (worst health) [39]
	Since the last assessment, how would you evaluate your patient’s Quality of Life (QoL)? Improved or remained unchanged; Slightly worsened (+1/+2 points); Significantly worsened (+3 points or more)	Use the difference between the initial and current EQ-5D-5L (LSS) scores

**Table 2:** Overview of the questions to be rated during the Delphi process for patients treated on demand.

Clinical Domain	Question with Related Answers	Definition
Bleeding episodes	What is the annualized bleeding rate (ABR)? Total ABR 0; Total ABR 1–2; Total ABR 3–4; Total ABR ≥ 5	ABR is calculated as the number of bleeding events divided by the duration of follow-up and multiplied by 365.25 [33]
Joint health	How many Problem Joints (PJs) does the patient currently present with? None; 1; 2–3; ≥4	A PJ is a joint with chronic pain and/or limited range of motion due to compromised joint integrity (e.g., chronic synovitis and/or hemophilic arthropathy), with or without persistent bleeding [34]
	How many Target Joints (TJs) does the patient currently present with? None; 1; ≥2	A TJ is a joint that has experienced three or more spontaneous bleeding episodes within a six-month period [35]
	Since the last assessment, how has the clinical evaluation of the patient’s joint status changed? Improved or remained approximately unchanged (at most +1 point); Moderately worsened (+2/+3 points); Significantly worsened (+4 points)	Clinical evaluation is based on the difference between the initial total Haemophilia Joint Health Score (HJHS) and the current total HJHS. The HJHS is a clinical assessment tool designed to detect early signs of haemophilic arthropathy. It is based on the evaluation of joint damage in the elbows, knees, and ankles, and includes both structural and functional components [37]
	Since the last assessment, how has the ultrasound evaluation of the patient’s joint status changed? Improved or remained unchanged; Moderately worsened (+1 point); Significantly worsened (+2 points or more)	Ultrasound evaluation is based on the difference between the initial total Haemophilia Early Arthropathy Detection with Ultrasound (HEAD-US) score and the current total HEAD-US score. The HEAD-US score is a semi-quantitative imaging tool designed to detect and monitor early joint changes in people with haemophilia using point-of-care musculoskeletal ultrasound. The HEAD-US system provides a simplified scale for detecting and quantifying early joint damage affecting the synovium, cartilage, and subchondral bone in the elbows, knees, and ankles. It allows for the evaluation of joint status and the monitoring of its progression as an indicator of arthropathy in patients with haemophilia [36]
	What is the annualized joint bleeding rate (AJBR)? Total AJBR 0; Total AJBR 1; Total AJBR 2; Total AJBR ≥ 3	The AJBR is calculated as the number of reported joint bleeding events divided by the duration of follow-up and multiplied by 365.25 [33]
QoL	How would you assess your patient’s current Quality of Life (QoL)? Very satisfactory (5–6 points); Moderately satisfactory (7–11 points); Poorly satisfactory (12–18 points); Not at all satisfactory (19–25 points)	Patient’s QoL is measured using the EQ-5D-5L (LSS) questionnaire. The EQ-5D-5L is a self-reported questionnaire that evaluates health status across five dimensions: mobility, self-care, usual activities, pain/discomfort, anxiety/depression. Each dimension has 5 levels of severity. The LSS is calculated by adding the numeric values from each of the five dimensions. The total score ranges from 5 (best health) to 25 (worst health) [39]

**Table 3:** Overview of the average weight and the consensus assigned to each clinical domain or section weight (W) for Haemophilia B patients in prophylaxis.

Clinical Domain	Median %	IQR
Pharmacokinetic	18	4
Bleeding episodes	32	2
Joint health	27	3
Adherence and QoL	23	6

**Table 4:** Overview of the average weight and the consensus assigned to each clinical domain or section weight (W) for Haemophilia A patients in prophylaxis.

Clinical Domain	Median %	IQR
Pharmacokinetic	19	5.5
Bleeding episodes	31	4.5
Joint health	29	4.75
Adherence and QoL	21	3.25

**Table 5:** Overview of the average weight and the consensus assigned to each clinical domain or section weight (W) for Haemophilia B patients on demand.

Clinical Domain	Median %	IQR
Bleeding episodes	40	0
Joint health	35	2
Adherence and QoL	25	6

**Table 6:** Overview of the average weight and the consensus assigned to each clinical domain or section weight (W) for Haemophilia A patients on demand.

Clinical Domain	Median %	IQR
Bleeding episodes	38	5.5
Joint health	37	2.5
Adherence and QoL	25	6

**Table 7:** Overview of the average weight assigned to each item domain (as a % of its domain) or specific question weight (w) for Haemophilia B patients in prophylaxis.

Clinical Domain	Question with Related Answers	Median %	IQR
Joint health	How many Problem Joints (PJs) does the patient currently present with? None; 1; 2–3; ≥4	13	2
	Since the start of the current treatment, the PJs have Absent or decreased; Remained stable; Increased	13	2
	How many Target Joints (TJs) does the patient currently present with? None; 1; ≥2	13	5
	Since the start of the current treatment, the TJs have: Absent or decreased; Remained stable; Increased	14	1
	Since the last assessment, how has the clinical evaluation of the patient’s joint status changed? Improved or remained approximately unchanged (at most +1 point); Moderately worsened (+2/+3 points); Significantly worsened (+4 points)	15	3
	Since the last assessment, how has the ultrasound evaluation of the patient’s joint status changed? Improved or remained unchanged; Moderately worsened (+1 point); Significantly worsened (+2 points or more)	19	3
	What is the annualized joint bleeding rate (AJBR)? Total AJBR 0; Total AJBR 1; Total AJBR 2; Total AJBR ≥ 3	13	4
Adherence and QoL	How would you rate your patient’s adherence to the prophylactic treatment? Very satisfactory (>90%); Satisfactory (between 80% and 90%); Poorly satisfactory (<80%)	32	5
	How would you assess your patient’s current Quality of Life (QoL)? Very satisfactory (5–6 points); Moderately satisfactory (7–11 points); Poorly satisfactory (12–18 points); Not at all satisfactory (19–25 points)	32	1
	Since the last assessment, how would you evaluate your patient’s Quality of Life (QoL)? Improved or remained unchanged; Slightly worsened (+1/+2 points); Significantly worsened (+3 points or more)	36	2

**Table 8:** Overview of the average weight assigned to each item domain (as a % of its domain) or specific question weight (w) for Haemophilia A patients in prophylaxis.

Clinical Domain	Question with Related Answers	Median %	IQR
Joint health	How many Problem Joints (PJs) does the patient currently present with? None; 1; 2–3; ≥4	11	5.25
	Since the start of the current treatment, the PJs have Absent or decreased; Remained stable; Increased	15	3
	How many Target Joints (TJs) does the patient currently present with? None; 1; ≥2	12	3.25
	Since the start of the current treatment, the TJs have: Absent or decreased; Remained stable; Increased	17	4.25
	Since the last assessment, how has the clinical evaluation of the patient’s joint status changed? Improved or remained approximately unchanged (at most +1 point); Moderately worsened (+2/+3 points); Significantly worsened (+4 points)	15	3.5
	Since the last assessment, how has the ultrasound evaluation of the patient’s joint status changed? Improved or remained unchanged; Moderately worsened (+1 point); Significantly worsened (+2 points or more)	17	3.5
	What is the annualized joint bleeding rate (AJBR)? Total AJBR 0; Total AJBR 1; Total AJBR 2; Total AJBR ≥ 3	13	5
Adherence and QoL	How would you rate your patient’s adherence to the prophylactic treatment? Very satisfactory (>90%); Satisfactory (between 80% and 90%); Poorly satisfactory (<80%)	33	4
	How would you assess your patient’s current Quality of Life (QoL)? Very satisfactory (5–6 points); Moderately satisfactory (7–11 points); Poorly satisfactory (12–18 points); Not at all satisfactory (19–25 points)	32	3.75
	Since the last assessment, how would you evaluate your patient’s Quality of Life (QoL)? Improved or remained unchanged; Slightly worsened (+1/+2 points); Significantly worsened (+3 points or more)	35	1.75

**Table 9:** Overview of the average weight assigned to each item domain (as a % of its domain) or specific question weight (w) for Haemophilia B patients on demand.

Clinical Domain	Question with Related Answers	Median %	IQR
Joint health	How many Problem Joints (PJs) does the patient currently present with? None; 1; 2–3; ≥4	18	1
	How many Target Joints (TJs) does the patient currently present with? None; 1; ≥2	19	3
	Since the last assessment, how has the clinical evaluation of the patient’s joint status changed? Improved or remained approximately unchanged (at most +1 point); Moderately worsened (+2/+3 points); Significantly worsened (+4 points)	20	4
	Since the last assessment, how has the ultrasound evaluation of the patient’s joint status changed? Improved or remained unchanged; Moderately worsened (+1 point); Significantly worsened (+2 points or more)	26	3
	What is the annualized joint bleeding rate (AJBR)? Total AJBR 0; Total AJBR 1; Total AJBR 2; Total AJBR ≥ 3	17	5

**Table 10:** Overview of the average weight assigned to each item domain (as a % of its domain) or specific question weight (w) for Haemophilia A patients on demand.

Clinical Domain	Question with Related Answers	Median %	IQR
Joint health	How many Problem Joints (PJs) does the patient currently present with? None; 1; 2–3; ≥4	18	3.25
	How many Target Joints (TJs) does the patient currently present with? None; 1; ≥2	19	2.75
	Since the last assessment, how has the clinical evaluation of the patient’s joint status changed? Improved or remained approximately unchanged (at most +1 point); Moderately worsened (+2/+3 points); Significantly worsened (+4 points)	23	5.25
	Since the last assessment, how has the ultrasound evaluation of the patient’s joint status changed? Improved or remained unchanged; Moderately worsened (+1 point); Significantly worsened (+2 points or more)	22	4
	What is the annualized joint bleeding rate (AJBR)? Total AJBR 0; Total AJBR 1; Total AJBR 2; Total AJBR ≥ 3	18	7

## Data Availability

The original contributions presented in this study are included in the article. Further inquiries can be directed to the corresponding author.

## Supplementary Materials

The following supporting information can be downloaded at: https://www.mdpi.com/article/10.3390/jcm15072533/s1, Supplementary Material S1: Step-by-step calculation of the composite Monitoring Tool score in a simulated patient with haemophilia A on EHL prophylaxis; Supplementary Material S2: User-Ready Monitoring Tool for Haemophilia A and B (Prophylaxis and On-Demand Versions); Supplementary Material S3: Delphi Participant Flow Diagram.
