# Supplementary material for: Integrating Clinical, Functional, and Patient-Reported Outcomes in Haemophilia Care: A Delphi-Based Consensus on a New Monitoring Tool
Source: J Clin Med. 2026 Mar 26;15(7):2533. doi: 10.3390/jcm15072533 (PMC13073016; doi:10.3390/jcm15072533)
Supplement: Supplementary file 1 [file jcm-15-02533-s001.zip › Supplementary_Material_S1.pdf]

SUPPLEMENTARY MATERIAL S1

Step-by-step calculation of the composite Monitoring Tool score in a simulated patient with haemophilia A on EHL prophylaxis

1. Clinical Scenario

The worked example refers to a 34-year-old man with severe haemophilia A receiving prophylaxis with recombinant factor VIII (rurioctocog alfa), 3000 IU every 3.5 days. He has been on this regimen for 3 years (previously treated with standard half-life rFVIII 3000 IU every other day).

At the current evaluation, the patient reports no total bleeding episodes (ABR = 0) and no target or problem joints. However, recent ultrasound assessment shows moderate worsening compared with the previous visit. Treatment adherence is rated as satisfactory (80–90%), while current quality of life is reported as poorly satisfactory with slight worsening since the last assessment.

2. Scoring Framework

For each item, the contribution to the composite score is calculated as:

Item contribution = $100 \times W \times w \times r$

Where:

- W = domain weight (sum across domains = 1)
- w = item weight within domain (sum within domain = 1)
- r = coded response value $\in [0,1]$

The multiplier 100 rescales the result to a 0–100 range.

Response coding:

- Four-point scale → $r = 0 ; 0.33 ; 0.67 ; 1$
- Three-point scale → $r = 0 ; 0.5 ; 1$

In all cases, 0 represents the most favourable clinical condition and 1 the least favourable.

3. Item-Level Calculation

Domain / Item	W	w	Selected Response	r	Contribution
**Pharmacokinetic** – Trough level satisfaction	0.19	1	Not very satisfactory	0.67	12.73
**Bleeding episodes** – ABR	0.31	1	0	0	0.00
**Joint health** – Problem Joints (current)	0.29	0.11	None	0	0.00
**Joint health** – Problem Joints (evolution)	0.29	0.15	Absent or decreased	0	0.00
**Joint health** – Target Joints (current)	0.29	0.12	None	0	0.00
**Joint health** – Target Joints (evolution)	0.29	0.17	Absent or decreased	0	0.00
**Joint health** – HJHS change	0.29	0.15	Improved or approx. unchanged	0.33	1.44
**Joint health** – HEAD-US change	0.29	0.17	Moderately worsened	0.67	3.30
**Joint health** – AJBR	0.29	0.13	0	0	0.00
**Adherence** – Prophylaxis adherence	0.21	0.33	Satisfactory (80–90%)	0.50	3.47

**QoL** – Current EQ-5D-5L	0.21	0.32	Poorly satisfactory (12–18)	0.67	4.50
**QoL** – QoL change	0.21	0.35	Slightly worsened	0.67	4.92

4. Total Score Computation

Domain subtotals:

- Pharmacokinetic: 12.73
- Bleeding episodes: 0.00
- Joint health: 4.74
- Adherence and QoL: 12.89

Total composite score = 12.73 + 0.00 + 4.74 + 12.89 = 30.36

Score categories:
0–25: Excellent
26–50: Suboptimal
51–75: Poor
76–100: Critical

The final score (30.36) falls within the Acceptable range.

5. Clinical Interpretation

This worked example illustrates how the Monitoring Tool captures early structural and patient-reported deterioration despite optimal bleeding control (ABR = 0). Moderate ultrasound worsening and reduced QoL contribute meaningfully to the composite score. The Suboptimal category should be interpreted as a structured clinical signal prompting comprehensive reassessment rather than as an automatic trigger for treatment escalation.
