# Supplementary material for: Integrating Clinical, Functional, and Patient-Reported Outcomes in Haemophilia Care: A Delphi-Based Consensus on a New Monitoring Tool
Source: J Clin Med. 2026 Mar 26;15(7):2533. doi: 10.3390/jcm15072533 (PMC13073016; doi:10.3390/jcm15072533)
Supplement: Supplementary file 1 [file jcm-15-02533-s001.zip › Supplementary_Material_S2.pdf]

SUPPLEMENTARY MATERIAL S2

User-Ready Monitoring Tool for Haemophilia A and B (Prophylaxis and On-Demand Versions)

This supplementary file provides clinician-facing printable forms for routine visits and pre-filled scoring sheets containing Delphi-derived weights for Haemophilia A and B in prophylaxis and on-demand settings.

This file contains four fully operational versions of the Monitoring Tool:

1. Haemophilia A – Prophylaxis
2. Haemophilia A – On-demand
3. Haemophilia B – Prophylaxis
4. Haemophilia B – On-demand

Each version includes:

- Clinician-facing questionnaire with explicit r coding
- Delphi-derived weights (W and w) pre-filled
- Step-by-step scoring sheet
- Interpretation ranges

Response Coding (r):

- For 4 response options → r = 0 ǀ 0.33 ǀ 0.67 ǀ 1
- For 3 response options → r = 0 ǀ 0.5 ǀ 1
- 0 = most favourable clinical condition ǀ 1 = least favourable clinical condition

HAEMOPHILIA A – PROPHYLAXIS

STEP 1 – Clinician Form

Pharmacokinetic (W = 0.18)
Trough level satisfaction (w = 1)

☐ Very satisfactory (r=0) ☐ Quite satisfactory (r=0.33)

☐ Not very satisfactory (r=0.67) ☐ Not at all satisfactory (r=1)

Bleeding episodes (W = 0.32)
Annualized Bleeding Rate (ABR) (w = 1)

☐ 0 (r=0) ☐ 1–2 (r=0.33)

☐ 3–4 (r=0.67) ☐ ≥5 (r=1)

Joint health (W = 0.27)
Number of Problem Joints (w = 0.11)

☐ None (r=0) ☐ 1 (r=0.33)

☐ 2–3 (r=0.67) ☐ ≥4 (r=1)

PJ evolution (w = 0.15)

☐ Absent/decreased (r=0) ☐ Stable (r=0.5) ☐ Increased (r=1)

Number of Target Joints (w = 0.12)

☐ None (r=0) ☐ 1 (r=0.5) ☐ ≥2 (r=1)

TJ evolution (w = 0.17)

☐ Absent/decreased (r=0) ☐ Stable (r=0.5) ☐ Increased (r=1)

HJHS change (w = 0.15)

☐ Improved/unchanged (r=0) ☐ Moderately worsened (r=0.33) ☐ Significantly worsened (r=1)

HEAD-US change (w = 0.17)

☐ Improved/unchanged (r=0) ☐ Moderately worsened (r=0.67) ☐ Significantly worsened (r=1)

AJBR (w = 0.13)

☐ 0 (r=0)

☐ 1 (r=0.33)

☐ 2 (r=0.67)

☐ ≥3 (r=1)

Adherence and QoL (W = 0.23)
Adherence (w = 0.33)

☐ >90% (r=0) ☐ 80–90% (r=0.5) ☐ <80% (r=1)

Current QoL (w = 0.32)

☐ Very satisfactory (r=0) ☐ Moderately satisfactory (r=0.33)

☐ Poorly satisfactory (r=0.67) ☐ Not at all satisfactory (r=1)

QoL change (w = 0.35)

☐ Improved/unchanged (r=0) ☐ Slightly worsened (r=0.5)

☐ Significantly worsened (r=1)

STEP 2 – Scoring Sheet

Domain	W	Item	w	Selected r	Contribution (100×W×w×r)
Pharmacokinetic	0.18	Trough level satisfaction	1		
Bleeding episodes	0.32	Annualized Bleeding Rate (ABR)	1		
Joint health	0.27	Number of Problem Joints	0.11		
Joint health	0.27	PJ evolution	0.15		
Joint health	0.27	Number of Target Joints	0.12		
Joint health	0.27	TJ evolution	0.17		
Joint health	0.27	HJHS change	0.15		
Joint health	0.27	HEAD-US change	0.17		
Joint health	0.27	AJBR	0.13		
Adherence and QoL	0.23	Adherence	0.33		
Adherence and QoL	0.23	Current QoL	0.32		
Adherence and QoL	0.23	QoL change	0.35		

STEP 3 – Total Score

Total Score = Sum of all item contributions (0–100).

Total Score =

STEP 4 – Interpretation

**Total Score**	**Interpretation**
0–25	Excellent (no treatment changes are necessary)
26–50	Suboptimal (treatment reassessment may be beneficial)
51–75	Poor (treatment re-evaluation is advised)
76–100	Critical (current management is likely inadequate; prompt revision recommended)

HAEMOPHILIA A – ON-DEMAND

STEP 1 – Clinician Form

Bleeding episodes (W = 0.40)
Annualized Bleeding Rate (ABR) (w = 1)

☐ 0 (r=0) ☐ 1–2 (r=0.33)

☐ 3–4 (r=0.67) ☐ ≥5 (r=1)

Joint health (W = 0.35)
Number of Problem Joints (w = 0.11)

☐ None (r=0) ☐ 1 (r=0.33)

☐ 2–3 (r=0.67) ☐ ≥4 (r=1)

PJ evolution (w = 0.15)

☐ Absent/decreased (r=0) ☐ Stable (r=0.5) ☐ Increased (r=1)

Number of Target Joints (w = 0.12)

☐ None (r=0) ☐ 1 (r=0.5) ☐ ≥2 (r=1)

TJ evolution (w = 0.17)

☐ Absent/decreased (r=0) ☐ Stable (r=0.5) ☐ Increased (r=1)

HJHS change (w = 0.15)

☐ Improved/unchanged (r=0) ☐ Moderately worsened (r=0.33)

☐ Significantly worsened (r=1)

HEAD-US change (w = 0.17)

☐ Improved/unchanged (r=0) ☐ Moderately worsened (r=0.67)

☐ Significantly worsened (r=1)

AJBR (w = 0.13)

☐ 0 (r=0) ☐ 1 (r=0.33)

☐ 2 (r=0.67) ☐ ≥3 (r=1)

Adherence and QoL (W = 0.25)
Current QoL (w = 0.32)

☐ Very satisfactory (r=0) ☐ Moderately satisfactory (r=0.33)

☐ Poorly satisfactory (r=0.67) ☐ Not at all satisfactory (r=1)

QoL change (w = 0.35)

☐ Improved/unchanged (r=0) ☐ Slightly worsened (r=0.5)

☐ Significantly worsened (r=1)

STEP 2 – Scoring Sheet

**Domain**	**W**	**Item**	**w**	**Selected r**	**Contribution (100×W×w×r)**
Bleeding episodes	0.40	Annualized Bleeding Rate (ABR)	1		
Joint health	0.35	Number of Problem Joints	0.11		
Joint health	0.35	PJ evolution	0.15		
Joint health	0.35	Number of Target Joints	0.12		
Joint health	0.35	TJ evolution	0.17		
Joint health	0.35	HJHS change	0.15		
Joint health	0.35	HEAD-US change	0.17		
Joint health	0.35	AJBR	0.13		
Adherence and QoL	0.25	Current QoL	0.32		
Adherence and QoL	0.25	QoL change	0.35		

STEP 3 – Total Score

Total Score = Sum of all item contributions (0–100).

Total Score =

STEP 4 – Interpretation

Total Score	Interpretation
0–25	Excellent (no treatment changes are necessary)
26–50	Acceptable (treatment reassessment may be beneficial)
51–75	Poor (treatment re-evaluation is advised)
76–100	Critical (current management is likely inadequate; prompt revision recommended)

HAEMOPHILIA B – PROPHYLAXIS

STEP 1 – Clinician Form

Pharmacokinetic (W = 0.18)
Trough level satisfaction (w = 1)

☐ Very satisfactory (r=0) ☐ Quite satisfactory (r=0.33)

☐ Not very satisfactory (r=0.67) ☐ Not at all satisfactory (r=1)

Bleeding episodes (W = 0.32)
Annualized Bleeding Rate (ABR) (w = 1)

☐ 0 (r=0) ☐ 1–2 (r=0.33)

☐ 3–4 (r=0.67) ☐ ≥5 (r=1)

Joint health (W = 0.27)
Number of Problem Joints (w = 0.11)

☐ None (r=0) ☐ 1 (r=0.33)

☐ 2–3 (r=0.67) ☐ ≥4 (r=1)

PJ evolution (w = 0.15)

☐ Absent/decreased (r=0) ☐ Stable (r=0.5) ☐ Increased (r=1)

Number of Target Joints (w = 0.12)

☐ None (r=0) ☐ 1 (r=0.5) ☐ ≥2 (r=1)

TJ evolution (w = 0.17)

☐ Absent/decreased (r=0) ☐ Stable (r=0.5) ☐ Increased (r=1)

HJHS change (w = 0.15)

☐ Improved/unchanged (r=0) ☐ Moderately worsened (r=0.33)

☐ Significantly worsened (r=1)

HEAD-US change (w = 0.17)

☐ Improved/unchanged (r=0) ☐ Moderately worsened (r=0.67)

☐ Significantly worsened (r=1)

AJBR (w = 0.13)

☐ 0 (r=0) ☐ 1 (r=0.33)

☐ 2 (r=0.67) ☐ ≥3 (r=1)

Adherence and QoL (W = 0.23)
Adherence (w = 0.33)

☐ >90% (r=0) ☐ 80–90% (r=0.5) ☐ <80% (r=1)

Current QoL (w = 0.32)

☐ Very satisfactory (r=0) ☐ Moderately satisfactory (r=0.33)

☐ Poorly satisfactory (r=0.67) ☐ Not at all satisfactory (r=1)

QoL change (w = 0.35)

☐ Improved/unchanged (r=0) ☐ Slightly worsened (r=0.5)

☐ Significantly worsened (r=1)

STEP 2 – Scoring Sheet

**Domain**	**W**	**Item**	**w**	**Selected r**	**Contribution (100×W×w×r)**
Pharmacokinetic	0.18	Trough level satisfaction	1		
Bleeding episodes	0.32	Annualized Bleeding Rate (ABR)	1		
Joint health	0.27	Number of Problem Joints	0.11		
Joint health	0.27	PJ evolution	0.15		
Joint health	0.27	Number of Target Joints	0.12		
Joint health	0.27	TJ evolution	0.17		
Joint health	0.27	HJHS change	0.15		
Joint health	0.27	HEAD-US change	0.17		
Joint health	0.27	AJBR	0.13		
Adherence and QoL	0.23	Adherence	0.33		
Adherence and QoL	0.23	Current QoL	0.32		
Adherence and QoL	0.23	QoL change	0.35		

STEP 3 – Total Score

Total Score = Sum of all item contributions (0–100).

Total Score =

STEP 4 – Interpretation

Total Score	Interpretation
0–25	Excellent (no treatment changes are necessary)
26–50	Acceptable (treatment reassessment may be beneficial)
51–75	Poor (treatment re-evaluation is advised)
76–100	Critical (current management is likely inadequate; prompt revision recommended)

HAEMOPHILIA B – ON-DEMAND

STEP 1 – Clinician Form

Bleeding episodes (W = 0.40)
Annualized Bleeding Rate (ABR) (w = 1)

☐ 0 (r=0) ☐ 1–2 (r=0.33)

☐ 3–4 (r=0.67) ☐ ≥5 (r=1)

Joint health (W = 0.35)
Number of Problem Joints (w = 0.11)

☐ None (r=0) ☐ 1 (r=0.33)

☐ 2–3 (r=0.67) ☐ ≥4 (r=1)

PJ evolution (w = 0.15)

☐ Absent/decreased (r=0) ☐ Stable (r=0.5) ☐ Increased (r=1)

Number of Target Joints (w = 0.12)

☐ None (r=0) ☐ 1 (r=0.5) ☐ ≥2 (r=1)

TJ evolution (w = 0.17)

☐ Absent/decreased (r=0) ☐ Stable (r=0.5) ☐ Increased (r=1)

HJHS change (w = 0.15)

☐ Improved/unchanged (r=0) ☐ Moderately worsened (r=0.33)

☐ Significantly worsened (r=1)

HEAD-US change (w = 0.17)

☐ Improved/unchanged (r=0) ☐ Moderately worsened (r=0.67)

☐ Significantly worsened (r=1)

AJBR (w = 0.13)

☐ 0 (r=0) ☐ 1 (r=0.33)

☐ 2 (r=0.67) ☐ ≥3 (r=1)

Adherence and QoL (W = 0.25)
Current QoL (w = 0.32)

☐ Very satisfactory (r=0) ☐ Moderately satisfactory (r=0.33)

☐ Poorly satisfactory (r=0.67) ☐ Not at all satisfactory (r=1)

QoL change (w = 0.35)

☐ Improved/unchanged (r=0) ☐ Slightly worsened (r=0.5)

☐ Significantly worsened (r=1)

STEP 2 – Scoring Sheet

**Domain**	**W**	**Item**	**w**	**Selected r**	**Contribution (100×W×w×r)**
Bleeding episodes	0.40	Annualized Bleeding Rate (ABR)	1		
Joint health	0.35	Number of Problem Joints	0.11		
Joint health	0.35	PJ evolution	0.15		
Joint health	0.35	Number of Target Joints	0.12		
Joint health	0.35	TJ evolution	0.17		
Joint health	0.35	HJHS change	0.15		
Joint health	0.35	HEAD-US change	0.17		
Joint health	0.35	AJBR	0.13		
Adherence and QoL	0.25	Current QoL	0.32		
Adherence and QoL	0.25	QoL change	0.35		

STEP 3 – Total Score

Total Score = Sum of all item contributions (0–100).

Total Score =

STEP 4 – Interpretation

Total Score	Interpretation
0–25	Excellent (no treatment changes are necessary)
26–50	Acceptable (treatment reassessment may be beneficial)
51–75	Poor (treatment re-evaluation is advised)
76–100	Critical (current management is likely inadequate; prompt revision recommended)
