# Supplementary material for: Integrating Clinical, Functional, and Patient-Reported Outcomes in Haemophilia Care: A Delphi-Based Consensus on a New Monitoring Tool
Source: J Clin Med. 2026 Mar 26;15(7):2533. doi: 10.3390/jcm15072533 (PMC13073016; doi:10.3390/jcm15072533)
Supplement: Supplementary file 1 [file jcm-15-02533-s001.zip › Supplementary_Material_S3.pdf]

SUPPLEMENTARY MATERIAL S3

Delphi Participant Flow Diagram

This supplementary figure presents a concise overview of the Delphi process conducted for the development and weighting of the Monitoring Tool. It summarizes the number of experts invited, those participating in Round 1, and those completing Round 2 separately for haemophilia A and haemophilia B.

Experts invited to participate<br>n = 20 haemophilia specialists

↓

Round 1 Participation

↓

Haemophilia A arm<br>Participated in Round 1<br>n = 16	Haemophilia B arm<br>Participated in Round 1<br>n = 12
Completed Round 2<br>n = 12	Completed Round 2<br>n = 9

Attrition between rounds was consistent with expected participation patterns in Delphi studies and, to the best of the authors' knowledge, was not attributable to methodological disagreement or scientific concerns.
